# 의사결정나무 분석을 이용한 한국 노인의 성별에 따른 건강관련 삶의 질 취약군 예측: 국민건강영양조사 자료 분석

**DOI:** 10.7586/jkbns.23.023

**Published:** 2024-02-14

**Authors:** Hee Sun Kim, Seok Hee Jeong

**Affiliations:** College of Nursing · Research Institute of Nursing Science, Jeonbuk National University, Jeonju, Korea; 전북대학교 간호대학 · 간호학연구소

**Keywords:** Decision trees, Aged, Quality of life, Men, Women, 의사결정나무, 노인, 삶의 질, 남성, 여성

## Abstract

**Purpose:**

The aim of this study was to predict the subgroups vulnerable to poorer health-related quality of life (HRQoL) according to gender in older adults.

**Methods:**

Data from 5,553 Koreans aged 65 or older were extracted from the Korea National Health and Nutrition Examination Survey. HRQoL was assessed using the EQ-5D tool. Complex sample analysis and decision-tree analysis were conducted using SPSS for Windows version 27.0.

**Results:**

The mean scores of the EQ-5D index were 0.93 ± 0.00 in men and 0.88 ± 0.00 in women. In men, poorer HRQoL groups were identified with seven different pathways, which were categorized based on participants’ characteristics, such as restriction of activity, perceived health status, muscle exercise, age, relative hand grip strength, suicidal ideation, the number of chronic diseases, body mass index, and income status. Restriction of activity was the most significant predictor of poorer HRQoL in elderly men. In women, the poorer HRQoL groups were identified with nine different pathways, which were categorized based on participants’ characteristics, such as perceived health status, restriction of activity, age, education, unmet medical service needs, anemia, body mass index, relative hand grip, and aerobic exercise. Perceived health status was the most significant predictor of poorer HRQoL in elderly women.

**Conclusion:**

This study presents a predictive model of HRQoL in older adults according to gender and can be used to detect individuals at risk of poorer HRQoL.

## 서론

### 1. 연구의 필요성

우리나라는 의료기술 발전, 건강한 생활방식 추구 등의 다양한 요인으로 평균 수명이 증가하고 있다. 특히 다른 연령층에 비해 65세 이상의 인구 비율이 급속하게 높아지고 있는 바, 2040년에는 노년층이 전체 인구의 34.4%를 차지하는 초고령 사회 진입이 예측된다[1]. 이러한 현상은 노인이 삶을 영위하는 과정에서 기대수명의 증가로 다양한 만성질환을 경험하고, 노년층의 건강유지에 따른 사회적 부담이 계속 증가하는 추세임을 의미한다[2]. 2020년도 국내 노인실태조사에 의하면 전체 노인 인구의 84%가 1개 이상의 만성질환을 가지고 있고, 13.5%에서 우울 증상을 호소하고 있어 만성질환이나 정신건강 문제로 고통받고 있는 노인 비율이 높은 것으로 보고되었다[3]. 더욱이, 노년기의 삶의 질에 있어서 건강문제가 차지하는 비중은 높기 때문에 노인의 건강관련 삶의 질 저하를 막고, 건강한 삶을 영위하는 것이 노년기의 중요한 과제 중 하나이다[3].

건강관련 삶의 질(Health-related quality of life, HRQoL)은 개인과 인구집단의 건강상태에 대한 대상자의 주관적인 평가를 확인하기 위해 측정되는 지표이다[4], 이러한 건강관련 삶의 질을 통하여 대상자의 일상기능, 신체적 및 정신적 안녕상태, 입원 혹은 사망률을 예측하여 건강서비스 요구를 추정할 수 있다[5]. 특히, 노인의 경우 건강관련 삶의 질은 노화의 핵심요소로서 노화에 따른 기능저하, 신체적, 기능적, 정신적 건강상태 및 증상관리와도 밀접하게 연관이 되어 있어[5,6], 노인의 운동능력, 자기관리, 일상활동, 통증, 우울 등의 영역에서 어느 정도 증상이나 제한을 경험하는지 살펴볼 필요가 있다[5,7].

노인의 건강관련 삶의 질에 대한 연구는 국내외 선행연구에서 전체 노인[8,9], 장기요양보험 재가급여노인[10], 교육수준이 낮은 노인[11], 고혈압 노인[12], 재가 고령노인[13] 등의 다양한 노인층을 대상으로 진행되어 왔다. 국외 체계적 문헌고찰에서는 노인의 건강관련 삶의 질의 영향요인으로 연령, 성별, 경제적 수준, 거주지역, 직업여부 등의 개인적 요인이 가장 밀접하게 연관이 되어 있는 것으로 밝혀졌고[14], 그 외 주관적 건강인지[15]와 동반질환 수, 만성질환 이환, 필요의료서비스 요구도 등의 건강관련 요인[11-13], 악력, 빈혈 등의 생리적 요인[9,16], 우울, 외로움, 자살생각 등의 정서적 요인[10,17], 흡연, 음주, 신체활동 등의 건강행태 등[11,12]의 다양한 요인들이 노인의 건강관련 삶의 질에 주요하게 영향을 미치는 것으로 분석되었다.

성별은 국가 전반적인 보건정책 수립 의사결정에 필수적인 역할을 하는 요인이기 때문에[18] 노인의 건강관련 삶의 질에도 중요한 역할을 하는 요인으로 여겨지고 있다. 기존 연구에서는 여성노인에서 신체적, 정신적 건강관련 삶의 질이 더 악화되었다는 보고들이 있지만[12,14,19] 여전히 성별 차이가 사회문화적 측면, 생태학적 측면과 연계하여 노인의 건강관련 삶의 질에 어떠한 역할을 하는지 모호한 실정이다. 이와 더불어, 성별 차이는 노인의 건강상태[20,21], 건강행태[22], 정신건강[23]에도 영향을 미치는 것으로 나타나 성별은 노인 인구집단을 대상으로 한 건강형평성 관련 정책수립을 위한 의사결정 시에 주요하게 고려되어야 할 필수적 요인이라 할 수 있다. 따라서, 무엇보다 기존의 선행연구결과에서 나타난 제한점을 보완하여 성별에 따라 건강관련 삶의 질을 저하시키는 요인들을 분석하는 것이 필요하다. 하지만 지금까지 국내 대규모 노인 인구를 대상으로 건강관련 삶의 질 연구에서 인구사회학적 특성, 건강관련 특성, 건강행태, 생리적 지표 등이 어떻게 노인의 건강관련 삶의 질에 영향을 미치는지 전반적으로 파악한 연구는 부족하다. 더욱이, 노년층에서 이러한 다양한 각각의 특성들이 성별에 따라 서로 연관성을 가지고 조합되어 나타나는 건강관련 삶의 질 취약군을 규명하는 연구는 찾아보기 힘든 실정이다.

의사결정나무 분석은 데이터마이닝 기법 중에 하나로서, 전체 집단의 방대한 자료 내에 존재하는 관계와 규칙들이 나무구조에 의한 추론규칙에 의하여 시각적으로 모형화되기 때문에 상대적으로 어떠한 요인이 특정 상태를 분류하는데 영향을 주는지 확인할 수 있다[24]. 또한 분류된 집단의 특성에 대해 정확한 분석과 예측이 가능하기 때문에 건강상태에 대한 고위험군 혹은 취약군의 패턴을 파악하여 예측모델을 형성하는데 유용하게 활용된다[15,25].

따라서, 노인의 특성과 관련된 다양한 변수들이 포함된 대표성이 있는 국민건강영양조사 자료를 이용하여 한국 노인을 대상으로 성별에 따른 건강관련 삶의 질 정도를 파악하고, 성별에 따라서 건강관련 삶의 질 취약군을 예측분석하는 연구가 필요하다 하겠다. 이에 본 연구에서는 국내 65세 이상의 노인을 대상으로 의사결정나무 분석을 통해 성별에 따른 건강관련 삶의 질 취약군을 예측할 수 있는 모형을 구축하고자 한다.

### 2. 연구의 목적

본 연구의 목적은 제7기(2016～2018년)와 제8기 1차(2019년) 국민건강영양조사자료를 활용하여 우리나라 65세 이상의 노인을 대상으로 성별에 따라서 일반적 특성, 생리적 지표, 건강행태, 정신건강 및 건강관련 삶의 질 정도 차이를 분석하고, 최종적으로 성별에 따른 건강관련 삶의 질 취약군을 규명하기 위함이다.

## 연구 방법

### 1. 연구 설계

본 연구는 2차 자료분석 연구로 국내 65세 이상의 노인의 인구사회학적 및 건강관련 특성, 생리적 특성, 건강행태, 정신건강 상태를 파악하고, 성별에 따른 건강관련 삶의 질 취약군을 규명하기 위한 서술적 조사연구이다.

### 2. 연구 대상

본 연구대상자는 제7기(2016-2018년)와 제8기 1차(2019년) 국민건강영양조사에 참여한 65세 이상의 노인이다. 제8기 2,3차(2020-2021년)에는 노인의 건강관련 삶의 질에 영향을 미칠 수 있는 변수인 악력이 조사되지 않아 제8기에서는 1차 자료만을 포함하였다. 국민건강영양조사의 표적 모집단은 다단계 층화집락 표본 추출법을 사용하여 표집한 대상자로서, 제7기와 제8기 1차에 참여한 전체 표본 수는 총 32,379명이었다. 이 중 65세 이상으로 구분된 대상자 수는 6,691명이었고 건강관련 삶의 질 척도인 EQ-5D 문항 결측치가 있는 631명, 최근 한달 간 와병상태인 507명을 제외하고 5,553명을 최종 연구대상자로 선정하였다.

### 3. 연구 도구

#### 1) 대상자의 일반적 특성

대상자의 인구사회학적 특성은 선행연구[10,14,15]에 기반하여 연령, 동거상태, 교육수준, 소득수준, 직업여부, 거주지역, 의료보험 종류를 조사하였다. 동거상태는 세대유형 중 1인 가족을 ‘혼자 산다’로, 그 외의 유형을 ‘가족이나 친척과 같이 산다’로 구분하였다. 소득수준은 월평균 개인소득 4분위수인 상, 중상, 중하, 하를 재분류하여 ‘상’, ‘중’, ‘하’로 구분하였으며, 직업은 직업 재분류 및 실업/비경제활동 상태를 재분류하여 직업 ‘유’와 ‘무’로 구분하였다.

대상자의 건강관련 특성은 선행연구[11-13,15]에 기반하여 만성질환, 주관적 건강인식, 필요의료 미충족을 조사하였다. 만성질환의 경우 고혈압, 이상지질혈증, 뇌졸중, 심근경색 또는 협심증, 골관절염 또는 류마티스관절염, 천식, 당뇨병, 암 중에서 현재 유병 중인 경우를 기준으로 0개, 1개, 2개, 3개 이상의 4군으로 분류하였다. 주관적 건강인식은 평소 본인의 건강에 대한 주관적 지각 정도에 따라 ‘좋음(매우 좋음, 좋음 포함)’, ‘보통’, ‘나쁨(매우 나쁨과 나쁨)’으로 재분류하여 구분하였다. 필요의료 미충족은 최근 1년 동안 병,의원 치료가 필요하였으나 받지 못한 경우 ‘미충족 있음’으로 구분하였다.

#### 2) 대상자의 생리적 특성

대상자의 생리적 특성은 선행연구[9,14,16]에 기반하여 체질량지수, 수축기혈압, 이완기혈압, 당화혈색소, 빈혈유무, 상대악력을 조사하였다. 체질량지수는 신장과 체중으로 계산하여 저체중(< 18.5 kg/m^2^), 정상(18.5 kg/m^2^-< 25 kg/m^2^), 비만(≥ 25 kg/m^2^)으로 분류하였다[26]. 수축기혈압과 이완기혈압은 1, 2, 3차 측정 결과를 기반으로 2, 3차 수축기혈압과 이완기혈압 평균값을 이용하였다. 수축기혈압은 140 mmHg 미만과 140 mmHg 이상으로, 이완기혈압이 90 mmHg 미만과 90 mmHg 이상으로 재분류하였다[27]. 당화혈색소는 6.5% 미만인 경우 정상으로 구분하였다[28]. 혈중 헤모글로빈 수치가 여성은 12 g/dL 미만, 남성은 13 g/dL 미만인 경우 빈혈이 있는 것으로 분류하였다[29]. 악력은 디지털 악력계를 사용하여 양손을 교대로 각각 3회씩 측정하였고, 본 연구에서는 양손의 평균값을 절대악력값으로 구한 후, 악력은 체중에 영향을 받기 때문에 절대악력값을 체중으로 나눈 상대악력값을 활용하였다. 그 후 군집분석(k-means analysis)을 통해 ‘악력 높은 군’과 ‘악력 낮은 군’ 2개로 재분류하였다.

#### 3) 건강행태와 정신건강

대상자의 건강행태는 선행연구[11,12,14]에 기반하여 흡연, 음주, 유산소운동, 근력운동, 걷기운동을 포함하였다. 흡연은 ‘비흡연자’, ‘현재 흡연자’, ‘과거 흡연자’로 구분하였고, 음주는 월간 음주율로 ‘평생 비음주 혹은 최근 1년간 월1잔 미만’과 ‘최근 1년간 월1잔 이상 음주’로 구분하였다. 유산소 운동여부는 일, 장소이동, 여가활동 영역에서 ‘일주일에 중강도 신체활동(숨이 약간 차거나 심장이 약간 빠르게 뛰는 활동)을 150분 이상 또는 고강도 신체활동(숨이 많이 차거나 심장이 매우 빠르게 뛰는 활동)을 75분 이상 또는 중강도와 고강도 신체활동을 혼합하여(고강도 1분 = 중강도 2분으로 계산) 각 활동에 상당한 시간을 실천하는 경우’를 유산소 운동 실천자로, 근력운동은 ‘최근 1주일 동안 팔굽혀 펴기, 윗몸 일으키기, 아령, 역기, 철봉 등의 근력운동을 한 날은 며칠입니까, 항목에 주 2,3,4,5일 이상’으로 응답한 경우를 근력운동 실천자로 구분하였다. 걷기운동은 ‘최근 1주일 동안 한번에 적어도 10분 이상 걸은 날은 며칠입니까, 항목과 이러한 날 중 하루 동안 걷는 시간은 보통 얼마나 됩니까, 항목에 주 5일 이상과 1일 30분 이상’으로 응답한 경우를 걷기운동 실천자로 구분하였다.

정신건강은 선행연구[10,17]에 기반하여 우울, 스트레스 인지, 자살생각을 조사하였다. 우울은 2주 이상 연속 우울감이 있는 경우 ‘예’로 구분하였으며, 스트레스 인지는 평소 일상생활 중에 스트레스를 ‘대단히 많이’ 혹은 ‘많이’ 느끼는 경우를 ‘스트레스 인지가 높은 군’으로, ‘조금 느끼는 경우’ 혹은 ‘거의 느끼지 않는 경우’를 ‘스트레스 인지가 낮은 군’으로 구분하였다. 자살생각은 최근 1년간 자살생각을 한 경우 ‘예’로 구분하였다.

#### 4) 건강관련 삶의 질

건강관련 삶의 질은 EQ-5D 지표와 세부항목인 운동능력, 자기관리, 일상생활, 통증/불편감, 불안/우울 항목으로 조사하였다. 각 항목에서 ‘문제 없음’ 1점, ‘중등도의 문제 있음’ 2점, ‘중증의 문제있음’ 3점으로 계산되고, EQ-5D 지표는 5개의 세부항목 점수에서 시간교환법으로 질 가중치를 적용한 계산값으로서, 가중 지표값으로 산출된다[4]. EQ-5D 지표값이 높을수록 건강관련 삶의 질이 높은 것을 의미한다. 본 연구에서 세부항목별로 ‘문제없음’, ‘중등도 혹은 중증 문제있음’의 2군으로 재분류하였다.

### 4. 자료 수집

본 연구는 국민건강영양조사 2016-2019년 총 4개년도 자료를 이용하였다. 국민건강영양조사는 대한민국에 거주하는 만 1세 이상의 국민을 모집단으로 하고 있으며 표본조사는 대상 인구의 연령, 성별, 지역, 동읍면, 주택유형을 기준으로 층화하고, 지역단위, 주거면적 비율, 가구주 학력비율 등의 층화집락기준을 활용한 다단계 층화 집락확률 추출법으로 자료가 수집되었다. 원시자료는 국민건강영양조사 홈페이지(https://knhanes.kdca.go.kr/knhanes/main.do)에서 사용자 정보 등록 후 사용승인을 받아 사용하였다.

### 5. 자료 분석

본 연구자료는 SPSS WIN 27.0 (IBM Corp., Armonk, NY, USA) 프로그램을 이용하였다. 연구변수들은 복합표본 설계 정보인 층화(strata), 집락(cluster), 4개년도 평균 가중치(weight)를 반영한 통계치를 산출하였다. 복합표본 자료에서는 가중치가 무응답의 경우에도 적용되기 때문에 결측값을 유효한 값으로 설정하고 분석하였다[30]. 복합표본 분석의 특성을 반영하여 가중되지 않는 빈도로 실수를, 가중치를 반영한 백분율과 평균 및 표준오차를 산출하였다. 대상자의 성별에 따른 일반적 특성 및 건강관련 삶의 질 차이를 분석하기 위하여 복합표본 교차분석을 이용하였다. 통계적 유의수준은 *p* < .05를 기준으로 하였다.

본 연구는 65세 이상 노인의 성별에 따른 건강관련 삶의 질 취약한 군을 규명하기 위하여 의사결정나무 분석을 이용하였다. 의사결정나무 분석 시 각 성별 그룹을 EQ-5D 지표값을 기준으로 K-means 군집분석을 통해 ‘삶의 질 높은 군’과 ‘삶의 질 낮은 군’ 2군으로 분류하였다. 의사결정나무 분석시에는 성별에 따른 건강관련 삶의 질 취약군을 가장 잘 예측하는 변인들의 조합 도출을 하기 위하여 Classification and regression tree (CRT) 방법으로 분석하였다. CRT는 자식노드내 종속변수 값들이 동질적이 되도록 부모마디로부터 자식마디가 2개로 분리되게 하는 방법으로서 최대한 노드 내 동질성의 극대화를 추구하고자 한다[24]. 성별에 따라서 대상자의 일반적 특성, 생리적 특성, 건강행태 및 정신건강 특성의 총 25개의 변수를 건강관련 삶의 질 취약군의 예측변수로 투입하였다. 본 연구에서 모형 설정값은 최대나무 깊이인 분류최대 분할수준 5, 분할될 부모노드 최소 크기 40, 자식노드 최소 크기 20, 향상의 최소 변화량 0.0001, 불순도 측정방법 Gini로 하였다[24,25]. 본 연구에서는 건강관련 삶의 질 취약군 모형의 안정성을 평가하기 위하여 10-fold 교차타당성 평가를 실시함으로써 구축된 모형예측력을 평가하는 과정을 총 10회 반복하여 평균 위험추정치 10개를 도출하고 이를 전체 자료로 구축한 모형의 위험추정치와 비교하였다[24,31].

### 6. 윤리적 고려

본 연구는 2차 분석한 연구로서, 통계자료 이용자 준수사항과 보안관련 내용에 대한 서약 후 개인식별 정보를 포함하지 않는 가상의 번호로 구분된 원시자료를 제공받았으므로 대상자의 익명성과 기밀성이 보장되었다. 본 연구수행 전 국민건강영양조사 홈페이지에서 사용승인을 받았으며, 전북대학교 생명윤리심의위원회에서 심의면제를 승인받은 후 연구를 진행하였다(JBNU 2023-10-001).

## 연구 결과

### 1. 대상자의 성별에 따른 일반적 특성 차이

대상자의 평균 연령은 72.84 ± 0.07세였으며, 전체 대상자 중 남성이 2,496명(44.5%), 여성이 3,057명(55.5%)이었다. 교육수준은 초등학교 이하 졸업생이 3,119명(55.4%)으로 가장 많았으며, 가족 혹은 친척과 동거하고 있는 경우는 4,315명(80.4%)이었다. 만성질환을 2개 이상을 가지고 있는 대상자는 2,585명(56.0%)이었고, 활동제한이 있는 대상자는 905명(15.1%)이었다.

성별에 따른 인구사회학적 특성 차이는 연령군(*p* < .001), 동거상태(*p* < .001), 교육수준(*p* < .001), 직업유무(*p* < .001), 의료보험 종류(*p* < .001)에 따라서 통계적으로 유의한 차이가 있었다. 80세 이상군에서 여성(61.9%)의 비율이 남성(38.1%)보다 더 많았고, 혼자 사는 군에서 여성(73.6%)의 비율이 남성(26.4%)보다, 초등학교 이하 졸업군에서 여성(70.1%)의 비율이 남성(29.9%)보다 높았다. 직업 없는 군에서는 여성(61.0%)의 비율이 남성(39.0%)보다 높았다.

대상자의 성별에 따른 건강관련 특성 차이는 의료보험 종류(*p* < .001), 만성질환 수(*p* < .001), 활동제한(*p* < .001), 주관적 건강인식(*p* < .001), 필요의료 미충족 여부(*p* < .001)에 따라서 통계적으로 유의한 차이가 있었다. 의료급여인 경우가 여성(66.5%)의 비율이 남성(33.5%)보다, 만성질환의 경우 3개 이상을 가진 군에서 여성(68.0%)의 비율이 남성(32.0%)보다 높았다. 활동제한이 있는 경우는 여성(60.9%)의 비율이 남성(39.1%)보다, 주관적 건강을 나쁘다고 인식하는 군에서는 여성(63.3%)의 비율이 남성(36.7%)보다 높았다. 필요의료 미충족이 있는 군의 경우에서는 여성(68.8%)의 비율이 남성(31.2%)보다 높았다(Table 1).

### 2. 대상자의 성별에 따른 생리적 지표, 건강행태 및 정신건강 차이

전체 대상자 중 1,617명(40.8%)이 비만이었으며, 평균 수축기혈압은 129.07 ± 0.27 mmHg, 평균 이완기혈압은 72.39 ± 0.16 mmHg, 평균 당화혈색소는 6.06 ± 0.01%이었다. 전체 대상자 중에서 743명(14.5%)이 빈혈을 가지고 있었고, 2,453명(46.9%)에서 상대악력이 낮은 것으로 나타났다. 대상자의 성별에 따른 생리적 지표 차이는 수축기혈압(*p* < .001), 빈혈(*p* < .001), 상대악력(*p* < .001)에 따라서 통계적으로 유의한 차이가 있었다. 수축기혈압 비정상군(≥ 140 mmHg)에서 여성(65.4%)의 비율이 남성(34.6%)보다 높았으며, 빈혈이 있는 경우 여성(59.4%)의 비율이 남성(40.6%)보다 높았다. 상대악력이 낮은 군에서 여성(58.9%)의 비율이 남성(41.1%)보다 높았다.

대상자의 건강행태에서는 전체 대상자 중 현재 흡연을 하고 있는 경우는 513명(9.4%), 1주 1잔 이상 음주를 하는 경우는 1,998명(36.5%)이었다. 대상자 중 유산소 운동을 하는 경우는 1,736명(31.7%)이었고, 근력운동은 976명(18.3%)이, 걷기운동은 949명(18.3%)이 실천하고 있었다. 성별에 따른 건강행태 차이는 흡연(*p* < .001), 음주(*p* < .001), 유산소 운동(*p* < .001), 근력운동(*p* < .001), 걷기(*p* = .026)에 따라서 통계적으로 유의한 차이가 있었다. 현재 흡연을 하는 경우 남성(87.4%)의 비율이 여성(12.6%)보다 높았으며, 1주 1잔 이상의 음주를 하는 경우 남성(72.2%)의 비율이 여성(27.8%)보다 높았다. 유산소 운동을 하는 경우 여성(58.7%)의 비율이 남성(41.3%)보다 높았고, 근력운동을 하는 경우 남성(71.0%)의 비율이 여성(29.0%)보다 높았다. 걷기 운동을 하는 경우 여성(52.1%)의 비율이 남성(47.9%)보다 높았다.

정신건강 측면에서는 2주간 우울감을 호소하는 대상자는 387명(13.5%)이었고, 주관적 스트레스를 높게 인지하고 있는 경우는 953명(17.2%)이었다. 자살생각을 해본 적이 있는 대상자는 190명(7.0%)이었다. 대상자의 성별에 따른 정신건강 차이는 우울(*p* < .001), 지각된 스트레스(*p* < .001)에 따라서 통계적으로 유의한 차이가 있었다. 2주 이상 연속 우울감이 있는 군에서 여성(66.1%)의 비율이 남성(33.9%)보다 높았으며, 지각된 스트레스가 높은 군에서 여성(70.5%)의 비율이 남성(29.5%)보다 높았다(Table 2).

### 3. 대상자의 성별에 따른 건강관련 삶의 질 차이

대상자의 건강관련 삶의 질 지표인 평균 EQ-5D index값은 0.90점 ± 0.00이었다. 건강관련 삶의 질 하부요인 중 중등도 혹은 중증의 문제를 가지고 있는 대상자는 운동 영역에서 1,946명(34.1%), 자가간호 영역에서 499명(8.4%), 일상생활 영역에서 952명(16.4%), 통증/불편감 영역에서 1,904명(33.2%), 불안/우울 영역에서 712명(11.9%)이었다. 대상자의 성별에 따른 건강관련 삶의 질 차이는 EQ-5D index값(*p* < .001)과 하부 영역 중 운동(*p* < .001), 자가관리(*p* < .001), 일상생활(*p* < .001), 통증/불편감(*p* < .001), 불안/우울(*p* < .001) 모두에 따라서 통계적으로 유의한 차이가 있었다(Table 2). 구체적으로는 EQ-5D index값은 여성이 평균 0.88 ± 0.00점으로 남성의 평균 0.93 ± 0.00점보다 낮았다. 운동능력, 자가관리, 일상생활능력에 중등도 혹은 중증의 문제가 있는 경우 여성(각 67.0%, 63.9%, 66.4%)의 비율이 남성(각 33.0%, 36.1%, 33.6%)보다 높았다. 통증/불편감과 불안/우울에 중등도 혹은 중증의 문제가 있는 경우 여성(각 67.0%, 67.0%)의 비율이 남성(각 33.0%, 33.0%)보다 높았다. 건강관련 삶의 질이 낮은 군의 경우 여성(67.5%)의 비율이 남성(32.5%)보다 높았다.

### 4. 대상자의 성별에 따른 건강관련 삶의 질 취약군 분류

**Figure 1.**
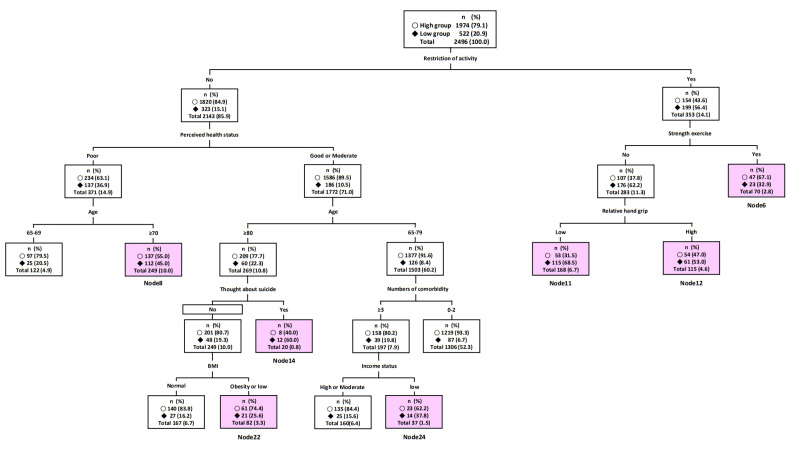
Decision-tree model to identify poorer health-related quality of life in elderly men.

**Figure 2.**
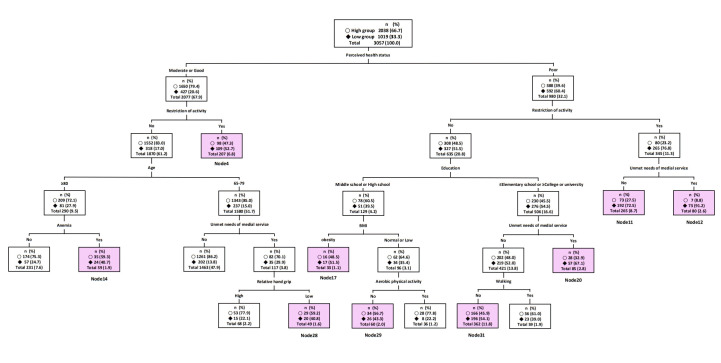
Decision-tree model to identify poorer health-related quality of life in elderly women.

본 연구에서는 국내 노인의 성별에 따른 건강관련 삶의 질 취약군을 규명하기 위하여 남성 노인과 여성 노인을 대상으로 각각 의사결정나무 분석을 실시하였다(Figure 1, 2). 본 연구에서 ‘건강관련 삶의 질 취약군’은 각 의사결정나무 분석에 투입된 전체 대상자의 평균을 나타내는 뿌리노드에서의 ‘낮은 건강관련 삶의 질 그룹의 비율’보다 해당 노드의 ‘낮은 건강관련 삶의 질 그룹의 비율이 높은 경우'로 정의하였다. 분석결과 뿌리노드에서 건강관련 삶의 질이 낮은 군의 비율은 남성에서 20.9%, 여성에서 33.3%로 나타났다. 이에, 의사결정나무의 각 끝 노드에서 ‘낮은 건강관련 삷의 질 그룹의 비율’이 남성은 20.9%보다 높은 경우, 여성은 33.3%보다 높은 경우를 ‘건강관련 삶의 질 취약군’으로 규정하였다.

의사결정나무 분석 결과, 남성노인의 끝노드 수는 총 11개가 산출되었고, 이 중 7개의 건강관련 삶의 질 취약군이 규명되었다. 여성노인의 끝노드 수는 총 14개가 산출되었고, 이 중 9개의 건강관련 삶의 질 취약군이 규명되었다. 성별에 따른 건강관련 삶의 질 취약군을 구체적으로 살펴보면 다음과 같다.

한국 남성노인의 건강관련 삶의 질 취약군은 7개로 규명되었으며(Table 3, Figure 1), 건강관련 삶의 질 취약군에 대한 의사결정나무 분지를 형성한 예측요인들은 총 9개로, 활동제한 여부, 주관적 건강인식, 근력운동 여부, 연령군, 상대악력, 자살생각 여부, 총 만성질환 수, 체질량지수, 소득수준이었다. 구체적으로 첫 번째 건강관련 삶의 질 취약군(노드 11)은 활동제한이 있으면서, 근력운동 미실천자면서, 상대악력이 낮은 그룹으로, 이 그룹의 68.5%가 낮은 건강관련 삶의 질을 보였다. 두 번째 건강관련 삶의 질 취약군(노드 14)은 활동제한이 없으면서, 주관적 건강상태를 보통 혹은 좋음으로 인식하고, 80세 이상이면서, 자살생각이 있는 그룹으로, 이 그룹의 60.0%가 낮은 건강관련 삶의 질을 보였다. 세 번째 건강관련 삶의 질 취약군(노드 12)은 활동제한이 있으면서, 근력운동 미실천자면서, 상대악력은 높은 그룹으로, 이 그룹의 53.3%가 낮은 건강관련 삶의 질을 보였다. 네 번째 건강관련 삶의 질 취약군(노드 8)은 활동제한이 없으면서, 주관적 건강상태를 나쁨으로 인식하고, 70세 이상인 그룹으로, 이 그룹의 45.0%가 낮은 건강관련 삶의 질을 보였다. 다섯 번째 건강관련 삶의 질 취약군(노드 24)은 활동제한이 없으면서, 주관적 건강상태를 보통 혹은 좋음으로 인식하고, 65-79세이면서, 만성질환을 3개 이상 가지고, 소득수준이 낮은(하) 그룹으로, 이 그룹의 37.8%가 낮은 건강관련 삶의 질을 보였다. 여섯 번째 건강관련 삶의 질 취약군(노드 6)은 활동제한이 있으면서, 근력운동 실천자인 그룹으로, 이 그룹의 32.9%가 낮은 건강관련 삶의 질을 보였다. 마지막 일곱 번째 건강관련 삶의 질 취약군(노드 22)은 활동제한이 없으면서, 주관적 건강상태를 보통 혹은 좋음으로 인식하고, 80세 이상이면서, 자살생각은 없고, 체질량지수가 비만 혹은 저체중인 그룹으로, 이 그룹의 25.6%가 낮은 건강관련 삶의 질을 보였다.

한국 여성노인의 건강관련 삶의 질 취약군은 9개로 규명되었으며(Table 3, Figure 2), 건강관련 삶의 질 취약군에 대한 의사결정나무 분지를 형성한 예측요인들은 총 10개로, 주관적 건강인식, 활동제한 여부, 연령군, 교육수준, 필요의료서비스 미충족 여부, 빈혈 여부, 체질량지수, 상대악력, 유산소운동 여부, 걷기 여부였다. 구체적으로 첫 번째 건강관련 삶의 질 취약군(노드 12)은 주관적 건강상태를 나쁨으로 인식하고, 활동제한이 있으며, 필요의료서비스가 미충족된 그룹으로, 이 그룹의 91.2%가 낮은 건강관련 삶의 질을 보였다. 두 번째 건강관련 삶의 질 취약군(노드 11)은 주관적 건강상태를 나쁨으로 인식하고, 활동제한이 있었으나, 필요의료서비스는 충족된 그룹으로, 이 그룹의 72.5%가 낮은 건강관련 삶의 질을 보였다. 세 번째 건강관련 삶의 질 취약군(노드 20)은 주관적 건강상태를 나쁨으로 인식하고, 활동제한은 없으며, 학력이 초졸 이하거나 대졸 이상이며, 필요의료서비스가 미충족된 그룹으로, 이 그룹의 67.1%가 낮은 건강관련 삶의 질을 보였다. 네 번째 건강관련 삶의 질 취약군(노드 31)은 주관적 건강상태를 나쁨으로 인식하고, 활동제한은 없으며, 학력이 초졸 이하 또는 대졸 이상이면서, 필요의료서비스가 충족되었으나, 걷기 미실천자인 그룹으로, 이 그룹의 54.1%가 낮은 건강관련 삶의 질을 보였다. 다섯 번째 건강관련 삶의 질 취약군(노드 4)은 주관적 건강상태를 보통 혹은 좋음으로 인식하고, 활동제한이 있는 그룹으로, 이 그룹의 52.7%가 낮은 건강관련 삶의 질을 보였다. 여섯 번째 건강관련 삶의 질 취약군(노드 17)은 주관적 건강상태를 나쁨으로 인식하고, 활동제한은 없으며, 학력이 중졸 또는 고졸로, 체질량지수 분류에서 비만인 그룹으로, 이 그룹의 51.5%가 낮은 건강관련 삶의 질을 보였다. 일곱 번째 건강관련 삶의 질 취약군(노드 29)은 주관적 건강상태를 나쁨으로 인식하고, 활동제한 없으면서, 학력이 중졸 또는 고졸이고, 체질량지수가 정상 또는 저체중이면서, 유산소 운동 미실천자 그룹으로, 이 그룹의 43.3%가 낮은 건강관련 삶의 질을 보였다. 여덟 번째 건강관련 삶의 질 취약군(노드 28)은 주관적 건강상태를 보통 또는 좋음으로 인식하고, 활동제한이 없으면서, 연령이 65-79세로, 필요의료서비스가 미충족되고, 상대악력이 낮은 그룹으로, 이 그룹의 40.8%가 낮은 건강관련 삶의 질을 보였다. 마지막 아홉 번째 건강관련 삶의 질 취약군(노드 14)은 주관적 건강상태를 보통 또는 좋음으로 인식하고, 활동제한이 없으면서, 80세 이상이고, 빈혈이 있는 그룹으로, 이 그룹의 40.7%가 낮은 건강관련 삶의 질을 보였다.

본 연구에서 남성 노인의 건강관련 삶의 질 취약군 규명을 위하여 구축된 모형의 위험추정값은 .18이었고, 10-fold 교차타당성 검증을 통한 평균 위험추정값은 .19로 거의 차이가 없었다. 여성 노인의 경우에도 취약군 규명을 위하여 구축된 모형의 위험추정값은 .25, 10-fold 교차타당성 검증결과의 평균 위험추정값은 .28로 거의 차이가 없었다. 이에, 남성 노인과 여성 노인의 건강관련 삶의 질 취약군 예측 모형의 안정성이 확보되었다.

## 논의

본 연구는 한국 노인의 성별에 따른 건강관련 삶의 질 정도 차이와 건강관련 삶의 질 취약군을 예측하는데 차이가 있는지를 확인하고자 시도되었다. 본 연구결과, EQ-5D index로 측정한 건강관련 삶의 질 수준은 여성 노인이 남성 노인에 비해 낮았다. 또한, 건강관련 삶의 질 5가지 하부 영역 즉 운동능력, 자가관리, 일상생활능력, 통증/불편감, 불안/우울 모두에서도 여성 노인이 남성 노인보다 중등도 혹은 중증의 문제가 있는 비율이 더 높은 것으로 밝혀졌다. 이는 기존 선행연구 결과들과 맥락을 같이 하는 것으로[17,19-21], 건강관련 삶의 질과 관련된 성별 불평등이 존재하는 것과 노인인구 집단에서 성별이 건강관련 삶의 질에 중요한 역할을 하고 있음을 시사하고 있다. 더욱이, 본 연구결과에서 독거상태, 저학력, 직업이 없는 경우, 의료급여, 필요의료서비스 미충족 등의 일반적 특성이나 빈혈, 상대악력 등의 생리적 특성, 우울이나 스트레스 등의 정신문제를 경험하는 비율이 여성 노인이 남성 노인보다 높은 것으로 나타나 이러한 취약한 특성들이 연계되어 여성 노인들이 지각하는 건강관련 삶의 질에 영향을 미칠 수 있음을 보여주고 있다.

본 연구결과 한국 노인의 건강관련 삶의 질 취약군을 예측할 수 있는 타당한 의사결정나무 모델이 제시되었다. 의사결정나무 분석을 활용한 예측모형에서, 나무의 주요분지를 형성하는 변인들은 결과변인에 대한 높은 영향력을 갖는데[24], 본 연구에서 남성 노인의 의사결정 모델에서 첫 번째 분지를 형성한 변인은 ‘활동제한 여부’, 두 번째 분지는 ‘주관적 건강인식’과 ‘근력운동 여부’였으며, 여성 노인에서는 첫 번째 분지를 형성한 변인이 ‘주관적 건강인식’, 두 번째 분지를 형성한 변인이 ‘활동제한 여부’로 나타나 유사한 양상을 보였다. 그러나 남성과 여성 노인의 건강관련 삶의 질 취약군을 규명하는데 분지를 형성한 변수는 남성 노인에서 9개, 여성 노인에서 10개로서, 구체적으로 살펴보면 주관적 건강인식, 활동제한 여부, 연령군, 상대악력, 체질량지수의 다섯 개 요인은 남성과 여성에서 분지를 형성하는 공통적인 변수였으나 이 외에 남성 노인에서는 추가적으로 근력운동 여부, 자살생각 여부, 총 만성질환 수, 소득수준이, 여성 노인에서는 교육수준, 필요의료서비스 미충족 여부, 빈혈여부, 유산소운동 여부, 걷기여부가 추가적인 분지형성 변수로 제시되어 노인 인구집단에서 성별에 따라 건강관련 삶의 질 취약층 분류가 차이점이 있음을 보여주고 있다.

본 연구결과 남성 노인과 여성 노인에서 공통적으로 제시된 요인들은 선행연구들[11,13,14]에서 건강관련 삶의 질의 주요 영향요인으로 제시되었기에, 성별을 고려하지 않은 일반적인 노인의 건강관련 삶의 질 향상 프로그램을 수혜받을 대상자들 선별 시 이들 공통요인들을 고려할 필요가 있다. 특히, 기존 노인을 대상으로 한 연구에서도 활동제한과 주관적 건강상태는 노인의 건강관련 삶의 질에 중요한 역할을 하는 변인으로 밝혀져, 건강관련 삶의 질 취약군을 분류할 때 노인들이 본인의 건강을 어떻게 인지하고 있는지를 파악하는 것이 중요하다 하겠다[10]. 그러나 본 연구의 주요 초점인 한국 노인의 각 성별에 특화된 맞춤형 건강관련 삶의 질 개선을 위해서는 남성과 여성의 차이를 반영할 필요가 있다. 예를 들면, 노인에서 뿐만 아니라 다른 연령층의 건강관련 삶의 질에 있어서도 운동은 신체적 건강[32] 뿐만 아니라 정신적 건강[33] 등 건강의 여러 부분에 긍정적인 영향을 주는 중요한 요소이다. 그러나 본 연구결과 각 성별에 따라 건강관련 삶의 질에 영향을 주는 운동의 종류에 차이가 제시되었는데 즉, 남성은 근력운동 여부가 분지형성 주요변수인 반면에 여성에서는, 근력운동이 아닌 유산소운동과 걷기가 취약군을 구성하는 분지의 주요변수로 확인되었다. 이는 근감소증을 가진 노인을 대상으로 한 문헌고찰에서 근력저항 운동이 근력에 미치는 영향이 남성 노인에서 더 크다는 결과와도 유사하며[22], 대학생의 신체활동과 운동 정체성, 정신건강에 대한 다집단 구조방정식모형을 제시한 선행연구[34]에서 신체활동 참여가 운동 정체성을 통해 정신건강에 영향을 미치는 방식이 남녀 대학생 집단 간에 다른 것으로 나타난 결과와 같은 맥락에서 이해할 수 있다. 또한 노인여성을 대상으로 아쿠아로빅 유산소 운동중재의 효과를 검정한 선행연구[35]에서 아쿠아로빅 운동이 노인여성의 신체구성의 개선과 체력을 향상을 향상시킴과 동시에 여성 노인의 건강관련 삶의 질(SF-36) 하부 영역 중 신체통증과 일반적인 건강에 긍정적인 영향을 미치는 것으로 제시된 것은 본 연구에서 여성 노인의 건강관련 삶의 질 취약군을 예측하는 주요 변수로서 유산소 운동 여부가 제시된 결과를 뒷받침 한다. 그리고, 국내 교육수준이 낮은 노인의 경우 걷기가 건강관련 삶의 질에 영향을 미치는 주요한 변수로 나타났기 때문에[11] 인구사회학적 요소들과 결합하여 성별에 따라 걷기 효과의 차이점이 있는지를 확인할 필요도 있겠다.

본 연구결과 제시된 건강관련 삶의 질 취약군을 각 성별에 따라 구체적으로 논의하면 다음과 같다. 먼저 남성 노인에서 규명된 7개 취약군 중, 상위 3개의 취약군에서는 해당 그룹의 50%이상의 대상자들 즉, 68.5%(노드 11), 60.0%(노드 14), 53.3%(노드 12)가 낮은 건강관련 삶의 질을 보였기에, 여러 취약군 중에서도 우선적인 접근과 중재가 요구된다. 첫 번째 취약군과 세 번째 취약군은 활동제한이 있으면서, 근력운동 미실천자인 동일 가지에서 분지하였으나, 상대악력이 낮은 경우는 첫 번째 취약군(노드 11)으로 규명되었으며, 상대악력이 높은 경우는 세 번째 취약군(노드 12)으로 규명되었다. 메타분석에 의하면[36], 근력운동은 저항운동과 같이 시행될 때 노인 대상자의 근감소증 발생을 낮추어주는 효과적인 중재로 밝혀졌기 때문에 노인에서 근력운동은 중요한 요인으로 작용될 수 있다. 더욱이 이들 그룹들에서는 활동의 제한이 있으므로 가능한 활동범위 내에서 실천할 수 있는 근력 증진을 위한 접근이 이루어져야 할 필요가 있다. 이때 메타분석 결과 노인의 근력에 유의한 영향을 주는 것으로 확인된 탄력밴드를 이용한 저항운동 등을 고려해볼 수 있겠다[37]. 또한, 악력은 노인에서 신체적 노쇠 뿐만 아니라 장애, 사망률을 예측할 수 있는 지표이면서 건강관련 삶의 질에 영향을 주는 주요한 요인으로 밝혀진 바[9,38], 남성 노인에서 첫 번째 취약군의 경우에는 근력운동과 함께 악력을 향상시킬 수 있는 중재가 함께 이루어져야 하겠다. 남성 노인의 두 번째 취약군은 활동제한이 없으면서, 주관적 건강상태를 보통 혹은 좋음으로 인식하고, 80세 이상이면서, 자살생각이 있는 그룹으로, 이 그룹의 60.0%에서 건강관련 삶의 질이 낮았다. 이 그룹은 활동제한이 없고 주관적 건강상태도 나쁘지 않으나, 연령이 80세 이상으로 노인 중에서도 고령노인(old-old)[4]에 해당되며, 특히 자살생각이 있어 건강관련 삶의 질이 낮은 두 번째 취약군으로 규명된 것으로 보인다. 같은 노인 대상자라고 해도 60대 노인과 80대 노인은 신체적, 심리적, 사회적 등 다양한 특징에서 동질집단으로 보기에는 다양성이 큰 것으로 제시되고 있는바[39], 본 연구결과를 통해 노인 대상자의 연령대의 특성 차이를 의사결정나무 분석을 통해 재확인할 수 있었다. 또한 자살은 건강관련 삶의 질의 하부 영역인 불안/우울과 밀접한 관련이 있는데, 한국의료패널조사 자료를 활용한 선행연구[40]에서 노인을 객관적 건강상태와 주관적 건강상태에 따라 구분한 4개 집단 모두에서 공통적으로 자살생각에 영향을 미치는 요인으로 불안과 우울 경험이 제시되었다. 따라서 자살생각이 있다고 응답한 이 그룹에 대해서는 건강관련 삶의 질 향상을 위한 접근뿐만 아니라 자살예방이 매우 시급하다. 따라서 이 그룹의 대상자들에게 정신건강관련 교육뿐만 아니라 남성 노인의 특성을 고려하여 사회적 연결망을 강화[41]하는 등의 남성 노인 맞춤형 자살예방프로그램이 제공되어야 하겠다. 이는 남성 노인의 건강관련 삶의 질을 향상시키는 데에도 긍정적인 영향을 줄 수 있을 것이다.

다음으로 여성 노인에서 규명된 9개 취약군 중 상위 6개의 취약군에서는 해당 그룹의 50% 이상의 대상자들이 낮은 건강관련 삶의 질을 보였다. 특히, 이들 중 노드 12(91.2%)와 노드 11(72.5%)은 남성의 첫 번째 취약군인 노드 11(68.5%)보다 건강관련 삶의 질이 낮은 대상자의 비율이 더 높아, 남성과 여성 전체에서 건강관련 삶의 질이 가장 낮은 첫 번째와 두 번째 취약군으로 확인되었다. 여성 노인에서의 첫 번째 및 두 번째 건강관련 삶의 질 취약군은 모두 주관적 건강상태를 나쁨으로 인식하고, 활동제한이 있는 가지에서 공통적으로 분지하였다. 이러한 결과는 기존 선행연구와 유사한 결과였다[42]. 그 이후에 필요의료서비스 미충족 여부에 따라, 미충족된 경우는 첫 번째 취약군(노드 12)으로 규명되었으며, 필요의료서비스가 충족된 경우에는 두 번째 취약군(노드 11)으로 규명되었다. 이 두 개의 취약군들은 모두 주관적 건강상태에 대하여 나쁘다고 인식하고 있으면서 활동의 제한도 있기 때문에, 좀 더 집중적인 의료서비스 제공이 요구되고 있다. 그럼에도 불구하고 이들 중 두 번째 취약군인 노드 11은 다행히도 필수의료서비스가 충족된다고 응답하고 있는 반면, 첫 번째 취약군인 노드 12는 필수의료서비스 조차 미충족되고 있다고 응답하고 있어 한국 여성노인들 중, 건강관련 삶의 질을 높이기 위한 서비스 뿐만 아니라 필수의료서비스에 대한 요구도의 우선순위가 높은 그룹으로 평가될 수 있다. 최근 건강형평성에 대한 중요성이 부각되고 있다. 세계보건기구(World Health Organization)는 모든 사람이 자신의 건강 잠재력을 최대한 이룰 수 있는 공평한 기회를 가져야한다고 건강형평성의 개념을 제시하였다[43]. 이는 사회적, 인구학적, 경제적, 또는 지역적 요인에 따른 모든 집단인 인구집단 간에 불공평한, 그리고 피하거나 고칠 수 있는 건강 격차가 존재하지 않아야 한다는 것[44]을 의미한다. 이러한 건강형평성의 측면에서 볼 때, 본 연구결과 여성 노인의 건강관련 삶의 질의 취약군 예측모형에서 주요 예측 변인으로 필수의료서비스 충족여부가 제시된 것은 매우 중요한 결과이다. 특히 본 연구결과 남성 노인에서는 나타나지 않고 여성 노인에서만 주요 예측요인으로 나타난 것은 더 큰 의미를 갖는데, 여성 노인의 전체 9개 건강관련 삶의 질 취약군 중 3개의 취약군(노드12, 노드 20, 노드 28)에서 필수의료가 미충족된 특성을 보여주고 있다. 한국에서 여성 노인은 남성 노인과 성별 역할에 많은 차이가 있어 왔으며, 사회경제적 지위가 다를 수 있고 또한 여성은 남성과의 생물학적 차이로 인해 이와 관련한 여성 특유의 건강문제가 발생할 수 있을 것으로 생각된다[18]. 따라서, 필수의료 미충족 여부가 여성 노인의 건강관련 삶의 질 취약군을 예측하는 모형의 주요 요인으로 제시된 결과를 근거로 하여, 여성 노인들의 건강서비스에 대한 접근성을 향상시킬 수 있는 방안을 마련하여 실행함으로써 여성 노인의 건강불평등을 해결할 필요가 있으며, 이를 통해 전체 노인 대상자들의 건강형평성 확보 및 건강관련 삶의 질 향상에 기여할 수 있을 것이다.

본 연구는 국민건강영양조사의 2차자료를 활용하였기에 노인의 건강관련 삶의 질에 영향을 주는 변수들 선정 및 측정 도구들의 타당도과 신뢰성 확보에 한계점을 가지고 있다. 그럼에도 불구하고, 노인의 건강관련 삶의 질 취약군을 규명하는데 있어 성별에 따른 다른 주요 요인들이 의사결정나무의 분지를 형성함을 확인함으로써 노인의 건강관련 삶의 질에 대한 접근 시 남성과 여성을 다르게 접근해야 함이 확인되었다. 이를 통해 한국 노인의 건강관련 삶의 질을 높이기 위한 간호 제공시, 성별에 따라 서비스와 중재를 먼저 제공해야 하는 취약군을 예측함으로써 제한된 자원의 활용에 있어 우선순위 설정의 근거를 제시하였다는데 본 연구의 의의가 있다.

## 결론

본 연구는 국민건강영양조사 자료를 활용한 의사결정나무 분석을 통해 성별에 따른 한국 노인의 건강관련 삶의 질 취약군 예측 모델을 제시하였으며, 한국 노인의 건강관련 삶의 질을 향상시키기 위한 간호 제공시, 간호서비스 우선적으로 제공해야 하는 남성 노인 취약군 7개와 여성 노인 취약군 9개를 규명하였다. 본 연구에서 남성 노인 취약군 예측요인들은 활동제한 여부, 주관적 건강인식, 근력운동 여부, 연령군, 상대악력, 자살생각 여부, 총 만성질환 수, 체질량지수, 소득수준의 총 9개였다. 여성 노인 취약군 예측요인들은 주관적 건강인식, 활동제한 여부, 연령군, 교육수준, 필요의료서비스 미충족 여부, 빈혈 여부, 체질량지수, 상대악력, 유산소운동 여부, 걷기 여부였다. 특히 본 연구결과 성별에 따라 제시된 예측모형은 성별의 특성을 반영한 취약군 선별 및 선별된 대상자들에 대한 맞춤형 간호서비스 제공을 가능케 함으로써 전체 노인 대상자들의 건강형평성 확보 및 건강관련 삶의 질 향상에 기여할 수 있을 것이다.

본 연구결과를 통해 다음과 같이 제언하고자 한다. 첫째, 지역사회 내 거주하는 노인들을 대상으로 활동제한, 주관적 건강상태 등을 확인할 수 있는 건강관련 삶의 질 취약군 선별 스크리닝 도구 개발을 제언한다. 둘째, 본 연구에서 사용된 측정도구들은 2차자료에서 사용된 도구들로서 추후 연구에서는 타당성과 신뢰성이 확보된 도구들을 사용할 것을 제언한다. 셋째, 성별에 따라 노인의 건강관련 삶의 질을 향상시킬 수 있는 차별화된 맞춤형 중재개발을 제언한다. 남성 노인을 대상으로 근력향상 운동중재 프로그램 개발과 남성 노인 맞춤형 자살예방 프로그램 개발 및 적용을 제어한다. 넷째, 여성 노인을 대상으로 유산소 운동에 초점을 둔 중재개발과 더불어 필수의료서비스 충족할 수 있는 건강서비스 접근성 향상 전략구축을 제언한다. 다섯째, 한국 노인의 건강형평성 보장 및 건강불평등 감소를 위하여 필수의료서비스 미충족군을 선별할 수 있는 예측모형 개발 연구의 실시를 제언한다.

## Figures and Tables

**Table 1.** Gender Differences in HRQoL according to the General Characteristics of Participants

Characteristics	Categories	n^†^ (%^‡^) or Mean ± SE^§^			Rao-Scott χ² (*p*)
		Total	Men	Women	
		5,533 (100%)	2,496 (44.5%)	3,057 (55.5%)	
Age (yr)	65∼69	1,793 (33.1)	822 (49.6)	971 (50.4)	21.05 (< .001)
	70∼79	2,809 (49.2)	1,275 (43.4)	1,534 (56.6)	
	≥ 80	951 (17.7)	399 (38.1)	552 (61.9)	
		72.84 ± 0.07	72.28 ± 0.09	73.29 ± 0.10	
Living status	Alone	1,238 (19.6)	328 (26.4)	910 (73.6)	219.21 (< .001)
	With family or relatives	4,315 (80.4)	2,168 (48.9)	2,147 (51.1)	
Education^∥^	≤ Elementary school	3,119 (55.4)	975 (29.9)	2,144 (70.1)	242.75 (< .001)
	Middle school	849 (15.6)	453 (54.7)	396 (45.3)	
	High school	979 (18.0)	638 (63.7)	341 (36.3)	
	≥ College or university	568 (11.0)	415 (72.5)	153 (27.5)	
Income status^∥^	High	1,445 (27.3)	647 (43.2)	798 (56.8)	0.97 (.380)
	Moderate	2,775 (48.5)	1,241 (44.9)	1,534 (55.1)	
	Low	1,305 (24.2)	594 (45.2)	711 (54.8)	
Occupation	Yes	1,905 (33.5)	1,041 (55.3)	864 (44.7)	132.92 (< .001)
	No	3,611 (66.5)	1,439 (39.0)	2,172 (61.0)	
Region	Dong (City)	4,042 (76.6)	1,838 (44.9)	2,204 (55.1)	0.96 (.329)
	Eup/Myun (Urban)	1,511 (23.4)	658 (43.4)	853 (56.6)	
Medical insurance^∥^	National health insurance	5,144 (93.9)	2,357 (45.4)	2,787 (54.6)	18.48 (< .001)
	Medical aid	350 (6.1)	121 (33.5)	229 (66.5)	
Number of comorbidities	0	232 (5.4)	112 (46.1)	120 (53.9)	47.56 (< .001)
	1	1,725 (38.6)	864 (50.6)	861 (49.4)	
	2	1,439 (30.9)	558 (38.6)	881 (61.4)	
	≥ 3	1,146 (25.1)	386 (32.0)	760 (68.0)	
Restriction of activity^∥^	Yes	905 (15.1)	353 (39.1)	552 (60.9)	14.69 (< .001)
	No	4,647 (84.9)	2,142 (45.5)	2,505 (54.5)	
Perceived health status	Good	1,246 (23.0)	693 (55.0)	553 (45.0)	50.51 (< .001)
	Moderate	2,755 (49.8)	1,231 (44.0)	1,524 (56.0)	
	Poor	1,552 (27.2)	572 (36.7)	980 (63.3)	
Unmet medical service needs^∥^	Yes	501 (8.8)	157 (31.2)	344 (68.8)	46.17 (< .001)
	No	4,999 (91.2)	2,305 (45.5)	2,694 (54.5)	

SE = standard error. ^†^non-weighted sample size; ^‡^weighted %; ^§^weighted mean & standard errors; ^∥^missing data included.

**Table 2.** Gender Differences in Physical Indicators, Health Behaviors, Mental Health, and Health-Related Quality of Life in Participants

Variables	Categories	n^†^ (%^‡^) or Mean ± SE^§^			Rao-Scott χ² or Wald F (*p*)
		Total	Men	Women	
		5,533 (100%)	2,496 (44.5%)	3,057 (55.5%)	
BMI (kg/m^2^)	< 18.5 (low)	147 (3.8)	83 (53.4)	64 (46.6)	2.33 (.098)
	18.5 ≤ BMI < 25 (normal)	2,215 (55.4)	1,053 (47.0)	1,152 (53.0)	
	≥ 25 (obesity)	1,617 (40.8)	725 (44.9)	892 (55.1)	
SBP (mmHg)^∥^	< 140 (normal)	4,157 (74.9)	2,000 (47.8)	2,157 (52.2)	66.41 (< .001)
	≥ 140 (abnormal)	1,392 (25.1)	494 (34.6)	898 (65.4)	
		129.07 ± 0.27	126.67 ± 0.35	131.00 ± 0.34	
DBP (mmHg)^∥^	< 90 (normal)	5,316 (96.0)	2,390 (44.4)	2,926 (55.6)	0.35 (.553)
	≥ 90 (abnormal)	233 (4.0)	104 (46.2)	129 (53.8)	
		72.39 ± 0.16	72.29 ± 0.22	72.48 ± 0.22	
HbA1c (%)^∥^	< 6.5 (normal)	4,206 (80.0)	1,911 (45.3)	2,295 (54.7)	0.01 (.938)
	≥ 6.5 (abnormal)	1,046 (20.0)	486 (45.1)	560 (54.9)	
		6.06 ± 0.01	6.05 ± 0.20	6.06 ± 0.02	
Anemia^∥^	Yes	743 (14.5)	327 (40.6)	416 (59.4)	8.33 (.004)
	No	4,509 (80.5)	2,070 (46.0)	2,439 (54.0)	
Relative hand grip strength^∥^	High	2,655 (53.1)	1,314 (49.6)	1,351 (50.4)	27.67 (< .001)
	Low	2,453 (46.9)	1,040 (41.1)	1,413 (58.9)	
Smoking^∥^	None or past smoker	5,014 (90.6)	2,037 (40.1)	2,977 (59.9)	307.28 (< .001)
	Current smoker	513 (9.4)	449 (87.4)	64 (12.6)	
Alcohol drinking^∥^ (per month in past 1 year)	None or less than	3,530 (63.5)	1,049 (28.7)	2,481 (71.3)	1088.17 (< .001)
	1 drink/month				
	More than 1 drink/week	1,998 (36.5)	1,438 (72.2)	560 (27.8)	
Aerobic physical activity^∥^	Yes	1,736 (31.7)	1,577 (41.3)	2,192 (58.7)	46.41 (< .001)
	No	3,769 (68.3)	899 (51.9)	837 (48.1)	
Strength exercise^∥^ (in past 1 week)	Yes	976 (18.3)	697 (71.0)	279 (29.0)	342.00 (< .001)
	No	4,552 (81.7)	1,789 (38.5)	2,766 (61.5)	
Walking^∥^ (in past 1 week)	Yes	949 (18.3)	448 (47.9)	501 (52.1)	5.01 (.026)
	No	4,554 (18.3)	2,023 (43.8)	2,531 (56.2)	
Depressive mood^∥^ (in past 2 weeks)	Yes	387 (13.5)	126 (33.9)	261 (66.1)	15.91 (< .001)
	No	2,404 (86.5)	1,121 (46.4)	1,283 (53.6)	
Perceived stress^∥^	High	953 (17.2)	283 (29.5)	670 (70.5)	162.64 (< .001)
	Low	4,567 (82.8)	2,201 (47.7)	2,366 (52.3)	
Suicidal ideation^∥^	Yes	190 (7.0)	81 (43.2)	109 (56.8)	0.14 (.711)
	No	2,600 (93.0)	1,165 (44.8)	1,435 (55.2)	
EQ-5D index		0.90 ± 0.00	0.93 ± 0.00	0.88 ± 0.00	247.61 (< .001)
Mobility	No problem	3,607 (65.9)	1,825 (50.4)	1,782 (49.6)	193.32 (< .001)
	Some or serious problem	1,946 (34.1)	671 (33.0)	1,275 (67.0)	
Self-care	No problem	5,054 (91.6)	2,306 (45.3)	2,748 (54.7)	17.49 (< .001)
	Some or serious problem	499 (8.4)	190 (36.1)	309 (63.9)	
Usual activities	No problem	4,601 (83.6)	2,157 (46.6)	2,444 (53.4)	65.93 (< .001)
	Some or serious problem	952 (16.4)	339 (33.6)	613 (66.4)	
Pain/discomfort	No problem	3,649 (66.8)	1,853 (50.2)	1,796 (49.8)	190.51 (< .001)
	Some or serious problem	1,904 (33.2)	643 (33.0)	1,261 (67.0)	
Anxiety/depression	No problem	4,841 (88.1)	2,257 (46.1)	2,584 (53.9)	39.61 (< .001)
	Some or serious problem	712 (11.9)	239 (33.0)	473 (67.0)	
HRQoL group	High	4,012 (73.6)	1,974 (48.8)	2,038 (51.2)	167.80 (< .001)
	Low	1,541 (26.4)	522 (32.5)	1,019 (67.5)	

SE = standard error; BMI = body mass index; SBP = systolic blood pressure; DBP = diastolic blood pressure; HbA1c = hemoglobin A1c; HRQoL = health-related quality of life. ^†^non-weighted sample size; ^‡^weighted %; ^§^weighted mean & standard errors; ^∥^missing data included.

**Table 3.** Gender-specific HRQoL Subgroup Analysis in Elderly

Group	HRQoL group	Node ID	Total	Risk group
			n (%)	n (%)
Men (n = 2,496)	Poor HRQoL			
		11	168 (6.7)	115 (68.5)
		14	20 (0.8)	12 (60.0)
		12	115 (4.6)	61 (53.0)
		8	249 (10.0)	112 (45.0)
		24	37 (1.5)	14 (37.8)
		6	70 (2.8)	23 (32.9)
		22	82 (3.3)	21 (25.6)
	Good HRQoL			
		Others	1,755 (70.3)	6.7% ～ 20.5%
Women (n = 3,057)	Poor HRQoL			
		12	80 (2.6)	73 (91.2)
		11	265 (8.7)	192 (72.5)
		20	85 (2.8)	27 (67.1)
		31	362 (11.8)	196 (54.1)
		4	207 (6.8)	109 (52.7)
		17	33 (1.1)	17 (51.5)
		29	60 (2.0)	26 (43.3)
		28	49 (1.6)	20 (40.8)
		14	59 (1.9)	24 (40.7)
	Good HRQoL			
		Others	1,857 (60.7)	13.8% ～ 39.0%

HRQoL = health-related quality of life.

## Data Availability

The data supporting the findings of this study are available in the article and appendix.

## References

[1] Statistics Korea (2021). Population status and outlook of the world and Korea reflecting future population estimates in 2021[Internet]. https://kostat.go.kr/board.es?mid=a10301020600&bid=207&act=view&list_no=420361.

[2] Park S, Nam JY (2022). Association between changes in multiple chronic conditions and health expenditures among elderly in South Korea: Korean longitudinal study of aging 2014–2018. Health Policy and Management.

[3] Lee YK, Kim SJ, Hwang NH, Lim JM, Ju BH, Namgoung EH

[4] Marten O, Brand L, Greiner W (2022). Feasibility of the EQ-5D in the elderly population: a systematic review of the literature. Quality of Life Research.

[5] Phyo AZZ, Freak-Poli R, Craig H, Gasevic D, Stocks NP, Gonzalez-Chica DA (2020). Quality of life and mortality in the general population: a systematic review and meta-analysis. BMC Public Health.

[6] Ohta R, Sato M (2021). The association between the self-management of mild symptoms and quality of life of elderly populations in rural communities: a cross-sectional study. International Journal of Environmental Research and Public Health.

[7] Blanco-Reina E, Valdellós J, Ocaña-Riola R, García-Merino MR, Aguilar-Cano L, Ariza-Zafra G (2019). Factors associated with health-related quality of life in community-dwelling older adults: a multinomial logistic analysis. Journal of Clinical Medicine.

[8] Moon H, Cha S (2022). Multilevel analysis of factors affecting health-related quality of life of the elderly. Journal of Korean Academy of Psychiatric and Mental Health Nursing.

[9] Back SH, Shin JE (2019). Association between grip strength and health-related quality of life in elderly Korean. Korean Journal of Sports Science.

[10] Kim JH, Park S (2023). A path analysis for health-related quality of life in long-term care insurance in-home service users. Research in Community and Public Health Nursing.

[11] Choi HY, Lee G (2020). Factors influencing health-related quality of life in the Korean seniors with lower education level: focusing on physical activity types. Korean Journal of Adult Nursing.

[12] Zheng E, Xu J, Xu J, Zeng X, Tan WJ, Li J (2021). Health-related quality of life and its influencing factors for elderly patients with hypertension: evidence from Heilongjiang province, China. Frontiers in Public Health.

[13] Sigeca F, Yip O, Mendieta MJ, Schwenkglenks M, Zeller A, Geest SD (2022). Factors associated with health-related quality of life among home-dwelling older adults aged 75 or older in Switzerland: a cross-sectional study. Health and Quality of Life Outcomes.

[14] Poursadeqiyna M, Arefi MF, Pouya AB, Jafari M (2021). Quality of life in health Iranian elderly population approach in health promotion: a systematic review. Journal of Education and Health Promotion.

[15] Ryu D, Sok S (2023). Prediction model of quality of life using the decision tree model in older adult single-person households: a secondary data analysis. Frontiers in Public Health.

[16] Wratsangka R, Putri RANH (2020). The importance of anemia and health-related quality of life in the elderly. Universa Medicina.

[17] Ko H, Park YH, Cho B, Lim KC, Chang SJ, Yi YM (2019). Gender differences in health status, quality of life, and community service needs of older adults living alone. Archives of Gerontology and Geriatrics.

[18] World Health Organization Gender and health [Internet]. https://www.who.int/news-room/questions-and-answers/item/gender-and-health.

[19] Lee S, Hong SH, Song HY (2020). Factors associated with health-related quality of life among older adults in rural South Korea based on ecological model. International Journal of Environmental Research and Public Health.

[20] Lee KH, Xu H, Wu B (2020). Gender differences in quality of life among community-dwelling older adults in low and middle-income countries: results from the study on global AGEing and adult health (SAGE). BMC Public Health.

[21] Kwon MG, Park MK, Kim HJ, Kim JI, Kim SA (2021). Factors influencing the muscle strength of the elderly without activity restrictions by gender. Journal of Korean Gerontological Nursing.

[22] Noh K, Park S (2023). Effects of resistance exercise on older individuals with sarcopenia: sex differences in humans. Exercise Science.

[23] Curran E, Rosato M, Ferry FR, Leavey G (2020). Prevalence and factors associated with anxiety and depression in older adults: gender differences in psychosocial indicators. Journal of Affective Disorders.

[24] Huh MI (2012). SPSS statistics classification analysis.

[25] Kim HS, Jeong SH (2021). Identification of subgroups with poor glycemic control among patients with type 2 diabetes mellitus: based on the Korean National Health and Nutrition Examination Survey from KNHANES VII (2016 to 2018). Journal of Korean Biological Nursing Science.

[26] Korean Society for The Study of Obesity (2022). Clinical Practice Guidelines for Obesity 2022 [Internet]. https://general.kosso.or.kr/html/?pmode=BBBS0001300003&page=1&smode=view&seq=1383&searchValue=&searchTitle=strTitle.

[27] Lee HY (2018). New definition for hypertension. Journal of the Korean Medicical Association.

[28] Korean Diabetes Association (2023). 2023 Clinical practice guidelines [Internet]. https://www.diabetes.or.kr/bbs/?code=guide&mode=view&number=1284&page=1&code=guide.

[29] Cappellinin MD, Motta I (2015). Anemia in clinical practice-definition and classification: does hemoglobin change with aging?. Seminars in Hematology.

[30] Korea Disease Control and Prevention Agency (2023). Korea National Health & Nutrition Examination Survey. Data Analysis guidline [Internet]. https://knhanes.kdca.go.kr/knhanes/sub03/sub03_06_02.do.

[31] Song Y, Ying LU (2015). Decision tree methods: applications for classification and prediction. Shanghai Archives of Psychiatry.

[32] Jo HH, Seo YH (2023). Effects of equipment pilates reformer exercise on changes in body composition and abdominal muscle strength in adult women. The Korean Journal of Growth and Development.

[33] Kang JC, Lee CH (2000). The effect of older adults' regular physical activity on mental health. The Korean Journal of Physical Education.

[34] Kim MJ, Yoo JI (2017). The relationships between physical activity participation, exercise identity, and mental health among university students. The Korean Journal of Physical Education.

[35] So WY, Hhong JY, Jun EJ, Choi DH, Kim KH (2010). Effects of aquarobics exercise on body composition, fitness and health related quality of life (SF-36) in elderly women. Journal of the Korean Gerontological Society.

[36] Shen Y, Shi Q, Nong K, Li S, Yue J, Huang J (2023). Exercise for sarcopenia in older people: a systematic review and networkmeta-analysis. Journal of Cachexai, Sarcopenia and Muscle.

[37] Yeun YR (2018). Resistance training on muscle strength among community-dwelling older adults: a systematic review and meta-analysis. Journal of the Korea Society of Computer and Information.

[38] Sayer AA, Kirkwood TB (2015). Grip strength and mortality: a biomarker of ageing?. Lancet.

[39] Jeon HS, Kahng SK (2012). Age differences in the predictors of medical service use between young-old and old-old: implications for medical service in aging society. Health and Social Welfare Review.

[40] Sung HY, Lee SK, Na JH (2021). A study on factors associated with suicidal ideation of older people: focused on the group comparisons by objective and subjective health status. Korean Journal of Social Welfare Research.

[41] Sim HI, Hong SI (2019). Moderating effect of social ties on the relationships between elder abuse, depression, and suicidal ideation. Korean Criminal Psychology Review.

[42] Son M (2022). Factors associated with levels of health-related quality of life in elderly women: secondary data analysis of the Korea National Health and Nutrition Examination Survey 2019. Korean Journal of Women Health Nursing.

[43] World Health Organization (2023). Guidance note on integrating health equity, gender equality, disability inclusion and human rights in WHO evaluations [Internet]. https://bit.ly/403GTbS.

[44] Cho SI (2015). Health equity. Journal of the Korean Medical Association.

